# Prediction of long-term mortality following hip fracture surgery: evaluation of three risk models

**DOI:** 10.1007/s00402-022-04646-4

**Published:** 2022-11-05

**Authors:** Julian Karres, Jan-Peter Eerenberg, Bart C. Vrouenraets, Gino M. M. J. Kerkhoffs

**Affiliations:** 1 Department of Orthopaedic Surgery, Amsterdam UMC, Meibergdreef 9, 1105 AZ Amsterdam, The Netherlands; 2 Department of Surgery, Tergooi Hospital, Hilversum, The Netherlands; 3 Department of Surgery, OLVG Hospital, Amsterdam, The Netherlands

**Keywords:** Hip fracture, Mortality, Risk prediction, Prognostic models

## Abstract

**Introduction:**

Several prognostic models have been developed for mortality in hip fracture patients, but their accuracy for long-term prediction is unclear. This study evaluates the performance of three models assessing 30-day, 1-year and 8-year mortality after hip fracture surgery: the Nottingham Hip Fracture Score (NHFS), the model developed by Holt et al. and the Hip fracture Estimator of Mortality Amsterdam (HEMA).

**Materials and methods:**

Patients admitted with a fractured hip between January 2012 and June 2013 were included in this retrospective cohort study. Relevant variables used by the three models were collected, as were mortality data. Predictive performance was assessed in terms of discrimination with the area under the receiver operating characteristic curve and calibration with the Hosmer–Lemeshow goodness-of-fit test. Clinical usefulness was evaluated by determining risk groups for each model, comparing differences in mortality using Kaplan–Meier curves, and by assessing positive and negative predictive values.

**Results:**

A total of 344 patients were included for analysis. Observed mortality rates were 6.1% after 30 days, 19.1% after 1 year and 68.6% after 8 years. The NHFS and the model by Holt et al. demonstrated good to excellent discrimination and adequate calibration for both short- and long-term mortality prediction, with similar clinical usefulness measures. The HEMA demonstrated inferior prediction of 30-day and 8-year mortality, with worse discriminative abilities and a significant lack of fit.

**Conclusions:**

The NHFS and the model by Holt et al. allowed for accurate identification of low- and high-risk patients for both short- and long-term mortality after a fracture of the hip. The HEMA performed poorly. When considering predictive performance and ease of use, the NHFS seems most suitable for implementation in daily clinical practice.

## Introduction

Hip fractures are associated with high mortality and represent a significant burden on both patients and healthcare systems [1–3]. As life expectancy continues to increase worldwide, so does the impact of hip fractures on our society [4, 5]. Patient characteristics related to high mortality following a fracture of the hip are well established and include age, gender, comorbidities and pre-fracture residency and mobility [6–9]. However, mortality risk assessment for the individual patient at the time of admission remains a challenge.

Risk prediction models can help identify hip fracture patients with a high or low probability of survival. Validated prognostic tools could be used to counsel patients and their families on prognosis, guide clinical decision making and inform surgeons and anaesthesiologists. Furthermore, prediction models can correct for differences in baseline variables and be of value when comparing quality of care between caregivers, hospitals or different time periods.

Several models for predicting mortality after hip fracture surgery have been developed. In our evaluation of six prediction models, the Nottingham Hip Fracture Score (NHFS) and the model by Holt and colleagues [10, 11] showed promising results but demonstrated a lack of fit, indicating a possible need for recalibration [12]. Our subsequently developed prediction model, the Hip fracture Estimator of Mortality Amsterdam (HEMA), demonstrated reasonable predictive performance but has not yet undergone external validation [13].

The NHFS, the model by Holt et al. and the HEMA all demonstrate adequate prediction of early mortality after hip fracture surgery in initial studies. For a risk model to be useful in clinical practice, however, external validation is required [14, 15]. Furthermore, the ability of these models to predict long-term mortality after a fracture of the hip has yet to be determined.

The aim of this study is to evaluate these risk prediction models for mortality in hip fracture patients and to compare their predictive performance for short- and long-term mortality.

## Patients and methods

This retrospective cohort study included all patients with a fractured hip admitted to the Tergooi hospital between January 2012 and June 2013. This hospital is a level 2 trauma centre located in Hilversum, The Netherlands. Surgical treatment was in accordance to current national guidelines. Patients with a periprosthetic femoral fracture or slipped capital femoral epiphysis were excluded, as were severe trauma patients (Injury Severity Score ≥ 16) and patients who were treated non-operatively. Three risk prediction models for mortality were evaluated.

### Nottingham Hip Fracture Score

The NHFS was developed in 2008 and underwent recalibration in 2012 to correct for overestimation of mortality in high-risk groups [11, 16]. The NHFS consists of seven variables and calculates the risk of mortality after hip fracture surgery using age, gender, serum haemoglobin, Abbreviated Mental Test Score (AMTS), whether the patient is living in an institution, the number of comorbidities and a history of malignancy. Between 1 and 4 points are attributed to each variable, with a maximum score of 10. Several studies evaluating the NHFS have proposed classification of patients into risk groups based on the aggregate score [17–19].

### Holt et al.

Holt and colleagues used data from the Scottish Hip Fracture Audit to analyse variables associated with mortality in hip fracture patients [10]. In their 2008 paper, the authors propose a prediction formula using preoperative variables and their logistic regression coefficients. The prediction model uses six variables to predict mortality after hip fracture surgery: age, American Society of Anesthesiologists (ASA) score, gender, pre-fracture residence, pre-fracture mobility and fracture type. Each variable has multiple subdivisions with according scores.

### Hip fracture estimator of mortality Amsterdam

We developed the HEMA in 2018 using nine variables to estimate mortality after hip fracture surgery: age, in-hospital fracture, signs of malnutrition, a history of myocardial infarction, congestive heart failure, renal failure, malignancy, current pneumonia and serum urea level [13]. Between 0.5 and 2 points are scored for each variable. Patients can be classified into low-, intermediate- and high-risk groups based on cumulative scores.

Data for the models were retrospectively collected from digital and paper medical records. Patient characteristics and comorbidities were recorded, as were laboratory results and operative variables. Individual mortality risk predictions by the three models were calculated for all patients. A history of cognitive impairment was used as a substitute for the AMTS, since this score was not available for the patients in our dataset. Any other missing values were not substituted but scored as negative in the calculation of individual mortality risks, reflecting the use of a prediction model in daily clinical practice.

The primary outcome of this study was mortality. Individual survival data were verified using the hospital’s administration records and through national databases. Thirty-day mortality was defined as death within 30 days following hip fracture surgery. To evaluate long-term survival and prediction, 1-year and 8-year mortality were assessed. Eight-year mortality was chosen as the endpoint for this study based on average life expectancies in the Netherlands. In 2012, life expectancy was 7.4 years for men and 9.1 years for women at age 81 [20]. This retrospective study and the use of clinical data was approved by the local ethical review board of our hospital. Due to the retrospective and observational nature of the research, individual informed consent was not required.

### Statistical analysis

Discrimination, calibration and clinical usefulness were analysed to assess predictive performance of the three models.

Discrimination is the ability of a model to separate patients who experienced the designated outcome from those who did not. It was evaluated using the Area Under the receiver operating characteristic Curve (AUC) [21]. Perfect discrimination is represented by an AUC of 1.00 whereas random predictions would result in an AUC of 0.50. When assessing mortality prediction, an AUC of 0.70 or higher is considered adequate discrimination, and an AUC ≥ 0.80 is considered good [22].

Calibration is a measure of how well predictions agree with observed outcomes. It was assessed using the Hosmer–Lemeshow goodness-of-fit test, which divides cases into prediction deciles and compares predicted versus observed mortality rates within these groups [23]. The Hosmer–Lemeshow test is significant when the differences are greater than would be expected by chance, indicating a lack of fit [23].

Clinical usefulness refers to a model’s capacity to aid in clinical decision making and depends on correct classification of patients [15, 24]. It was evaluated by determining low-, intermediate- and high-risk groups for each model based on cumulative scores and predicted mortality rates. Differences in mortality between risk groups were assessed with Kaplan–Meier curves, plotting survival after hip fracture surgery over time. Additionally, the positive predictive value (PPV) and negative predictive value (NPV) were evaluated for high-risk and low-risk groups, respectively. PPV is defined as the likelihood that a person with a positive result truly has the designated outcome; it is the probability of death for a patient in the high-risk group. NPV refers to the probability of survival for a patient in the low-risk group.

A *p* value of < 0.05 was considered statistically significant. All analyses were performed with SPSS Statistics, version 26.0 (IBM, NY, USA).

## Results

A total of 344 surgically treated patients with a fractured hip were included between January 2012 and June 2013. Patient characteristics and operative variables are described in Table 1. Median age was 81 years, 70.3% of patients were female, and the majority of patients had an ASA score of 2 (52.9%) or 3 (28.2%). Most patients were operated on the day following admission. Twenty-one patients (6.1%) died within 30 days following hip fracture surgery. Mortality rates were 19.1% after 1 year and 68.6% after 8 years (Table 1).

**Table 1:** Patient characteristics, operative variables and mortality rates

**Variables**		Missing data
Age		
In years, median (IQR)	81 (69–87)	–
Gender		
Female	242 (70.3)	–
Male	102 (29.7)	
ASA score		–
1	55 (16.0)	
2	182 (52.9)	
3	97 (28.2)	
4	10 (2.9)	
Time to surgery		
Same day	124 (36.0)	–
Next day	196 (57.0)	
Two days or more	24 (7.0)	
Anaesthesia		
General	188 (65.3)	56 (16.3)
Regional	100 (34.7)	
Mortality		
30-day	21 (6.1)	–
1-year	65 (19.1)	3 (0.9)
8-year	218 (68.6)	26 (7.6)

Values are *n* (%) unless otherwise stated

*IQR* interquartile range, *ASA* American Society of Anesthesiologists

Variables for the three prediction models are described in Table 2, as are their respective cut-off values and attributed points. Prevalence of the variables as a proportion of the total dataset is reported as well. The NHFS uses seven variables and has a subdivision for age, attributing 0 to 4 points per value. The model by Holt et al. uses six variables with 23 subdivisions, attributing either positive or negative values to two decimal places for each option. The HEMA uses nine variables with no further subdivision and attributes between 0 and 2 points. With all models, risk prediction as a percentage can be calculated with the cumulative point scores and the logistic regression formula (Table 2).

**Table 2:** Variables for risk prediction models, calculation methods and proportions in the dataset

Prediction model	Value	Score	Proportion *n* (%)
*NHFS*			
Age in years			
	< 66	0	73 (21.2)
	66–85	3	157 (45.6)
	≥ 86	4	114 (33.1)
Gender	Male	1	102 (29.7)
Haemoglobin	≤ 10 g dl^−1^	1	24 (7.0)
Cognitive impairment	Yes	1	64 (18.6)
Living in an institution	Yes	1	68 (19.8)
Number of comorbidities	≥ 2	1	116 (33.7)
Malignancy	Yes	1	59 (17.2)
Predicted 30-day mortality (%)	100/1 + e^[5.0122 − (NHFS score × 0.481)]		
*Holt et al.*			
Age in years	< 60	0	43 (12.5)
	60–69	0.58	48 (14.0)
	70–79	1.24	65 (18.9)
	80–89	1.74	128 (37.2)
	≥ 90	1.96	60 (17.4)
ASA score	1 or 2	0	237 (68.9)
	3	0.80	97 (28.2)
	4 or 5	1.62	10 (2.9)
Gender	Male	0	242 (70.3)
	Female	− 0.65	102 (29.7)
Pre-fracture residence	Own home	0	172 (50.0)
	Long-term care	0.53	58 (16.9)
	Rehabilitation	0.53	7 (2.0)
	Acute hospital ward	0.59	4 (1.2)
Pre-fracture mobility	No aids	0	261 (75.9)
	One aid	− 0.02	8 (2.3)
	Two aids / frame	0.07	62 (18.0)
	Requires accompaniment	0.24	9 (2.6)
	Unable to walk	0.45	4 (1.2)
Fracture type	Intracapsular	0	115 (33.4)
	Extracapsular	0.12	202 (58.7)
	Subtrochanteric	0.28	23 (6.7)
	Pathological	1.32	4 (1.2)
Predicted 30-day mortality (%)	100/1 + *e*^[4.79 – Holt et al. score]		
*HEMA*			
Age in years	≥ 85	1	128 (37.2)
In-hospital fracture	Yes	2	4 (1.2)
Signs of malnutrition	Yes	2	2 (0.6)
Myocardial infarction	Yes	1	17 (4.9)
Congestive heart failure	Yes	1	20 (5.8)
Current pneumonia	Yes	2	2 (0.6)
Renal disease	Yes	1	20 (5.8)
Malignancy	Yes	1.5	59 (17.2)
Serum urea	> 9 mmol/L	0.5	30 (8.7)
Predicted 30-day mortality (%)	100/1 + *e*^[3.823 – HEMA score]		

*NHFS* Nottingham Hip Fracture Score, *ASA* American Society of Anesthesiologists, *HEMA* Hip fracture Estimator of Mortality Amsterdam

### Discrimination

Table 3 displays performance measures of the three prognostic models. There was a statistically significant difference in discrimination between the model by Holt et al. and the HEMA for 30-day mortality (*p* = 0.039). There were no significant differences between the NHFS and the other two models for 30-day mortality. Discrimination for 1-year mortality did not differ significantly between the three prediction models. Discriminative performances of both the NHFS and the model by Holt et al. were significantly better than that of the HEMA when predicting 8-year mortality (*p* < 0.001).

**Table 3:** Performance indicators for prediction models of mortality after hip fracture surgery

	Discrimination	Calibration	Clinical usefulness	
	AUC	H–L	PPV (%)	NPV (%)
30-day mortality				
NHFS	0.80 (0.72–0.87)	*p* = 0.164	14.9	100.0
Holt et al.	0.86 (0.78–0.94)	*p* = 0.293	17.2	99.1
HEMA	0.74 (0.63–0.85)	***p* = 0.025**	17.9	96.8
1-year mortality				
NHFS	0.76 (0.70–0.82)	*p* = 0.322	40.5	95.0
Holt et al.	0.79 (0.73–0.84)	*p* = 0.709	40.9	95.4
HEMA	0.73 (0.66–0.80)	*p* = 0.331	48.7	89.1
8-year mortality				
NHFS	0.90 (0.86–0.93)	*p* = 0.063	98.6	72.4
Holt et al.	0.89 (0.85–0.93)	*p* = 0.413	94.5	74.7
HEMA	0.79 (0.75–0.84)	***p* < 0.001**	97.4	40.5

Values in parentheses are 95% confidence intervals. AUC, area under the curve. H–L, Hosmer–Lemeshow goodness-of-fit test. Bold typeface indicates statistically significant results (*p* < 0.05). PPV, positive predictive value is the probability of death for a patient in the high-risk group. NPV, negative predictive value is the probability of survival for a patient in the low-risk group. NHFS, Nottingham Hip Fracture Score. HEMA, Hip fracture Estimator of Mortality Amsterdam

### Calibration

The NHFS and the model by Holt et al. demonstrated adequate calibration for 30-day, 1-year and 8-year mortality. The Hosmer–Lemeshow goodness-of-fit test was significant for the HEMA in 30-day and 8-year mortality predictions, indicating a lack of fit (Table 3).

### Clinical usefulness

**Fig. 1:**
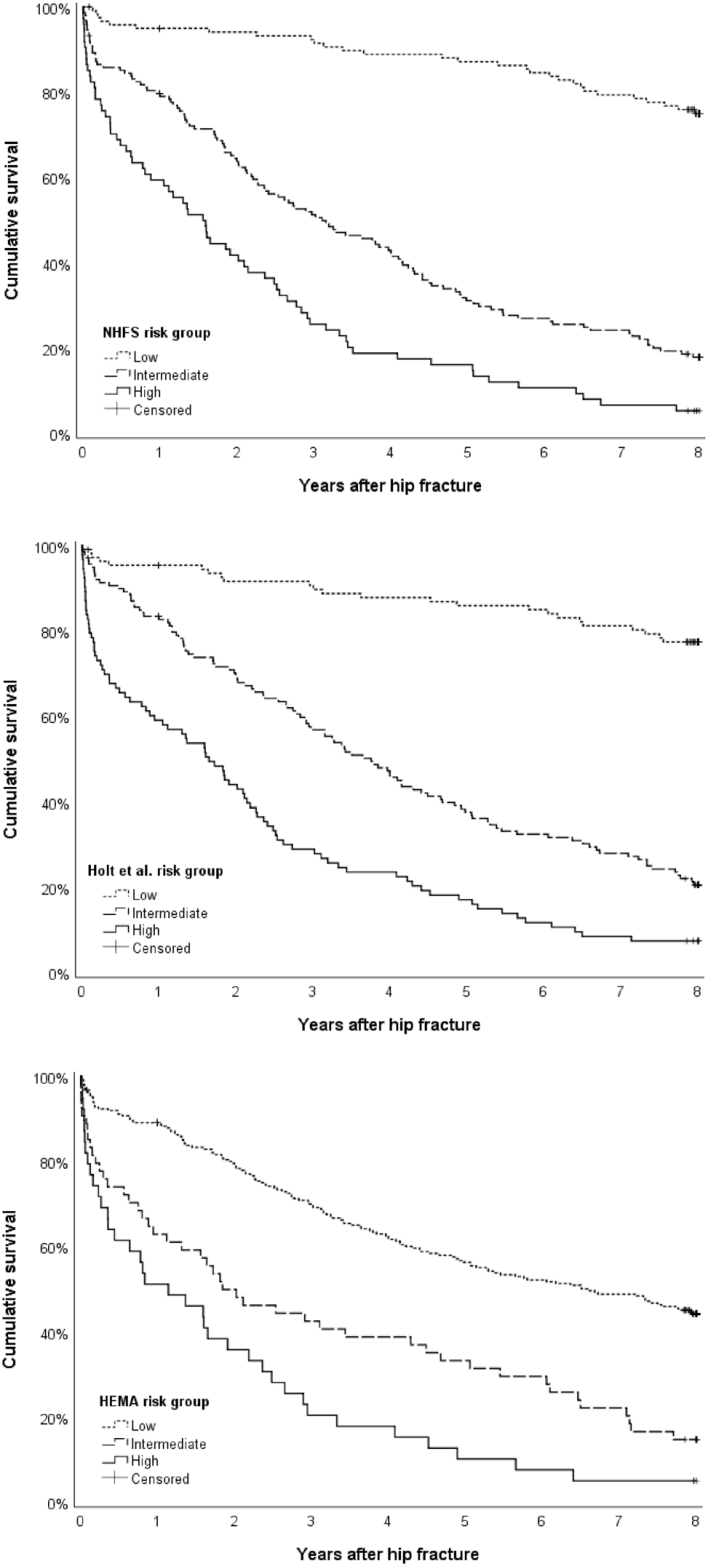
Kaplan–Meier curves demonstrating survival probabilities of patients in low-, intermediate-, and high-risk groups as classified by **a** the Nottingham Hip Fracture Score (NHFS), **b** the risk model by Holt et al., and **c** the Hip fracture Estimator of Mortality Amsterdam (HEMA)

Table 4 describes classification of risk groups using cut-off values for cumulative scores and predicted mortality rates. Risk groups and their proportion in the dataset are described (Table 4). Positive predictive values for the high-risk groups and negative predictive values for the low-risk groups are reported. Death rates in the high-risk group ranged between 14.9 and 17.9% after 30 days and between 40.5 and 48.7% after 1 year. At 8 years, mortality rates in the high-risk group resulted in a PPV of 94.5–98.6%. Survival in the low-risk group was 96.8–100% after 30 days, 89.1–95.4% after 1 year and 40.5–74.7% after 8 years (Table 3). The Kaplan–Meier curves displayed in Fig. 1 graph cumulative survival for low-, intermediate-, and high-risk groups for each risk prediction model over the 8-year period.

**Table 4:** Clinical usefulness of risk groups based on cumulative scores and proportions in the dataset

Prediction model	Cumulative score	Predicted 30-day mortality rate (%)	Proportion *N* (%)
NHFS risk group			
Low	≤ 3	≤ 2.7	121 (35.2)
Intermediate	4–5	4.4–6.9	149 (43.3)
High	≥ 6	≥ 10.7	74 (21.5)
Holt et al. risk group			
Low	< 1	< 2.2	111 (32.3)
Intermediate	1–2	2.2–5.8	140 (40.7)
High	> 2	> 5.8	93 (27.0)
HEMA risk group			
Low	≤ 1	≤ 5.6	251 (73.0)
Intermediate	1.5–2	8.9–13.9	54 (15.7)
High	≥ 2.5	> 21.0	39 (11.3)

*NHFS* Nottingham Hip Fracture Score, *HEMA* Hip fracture Estimator of Mortality Amsterdam

## Discussion

This external validation study evaluates the performance of three risk prediction models mortality following hip fracture surgery: the NHFS, the model by Holt et al. and the HEMA. Thirty-day, 1-year and 8-year mortality were assessed.

When predicting 30-day mortality, the NHFS and the model by Holt et al. demonstrated good discriminative ability. The HEMA showed adequate discrimination, but performed significantly worse than the model by Holt et al. Furthermore, the HEMA had a significant lack of fit. At 30 days, positive predictive values were limited for all risk models. Negative predictive values were high, particularly for the NHFS; all low-risk patients survived after 30 days.

One-year mortality prediction was similar between the three models. Discrimination was adequate without significant differences, and all models demonstrated sufficient calibration. While the PPV increased, the NPV decreased only slightly.

Prediction of 8-year mortality by the NHFS and the model by Holt et al. resulted in good to excellent discrimination and adequate calibration. The HEMA performed poorly with inferior discriminative abilities and a significant miscalibration. The PPV was high for all models. The NPV decreased most notably in the HEMA where only 40.5% of low-risk patients survived after 8 years. In contrast, 8-year survival rates of low-risk patients were 72.4% and 74.7% for NHFS and the model by Holt et al., respectively.

The NHFS has been validated in numerous papers for both 30-day and 1-year mortality [25–29]. It is the most commonly studied prediction model for mortality in hip fracture patients and frequently demonstrates adequate predictive performance in line with our findings, although several authors have reported on the need for recalibration [16, 18, 30]. With only seven variables, the NHFS is easy to use and even available as a smart phone application [31]. The inclusion of the Abbreviated Mental Test Score makes implementation slightly more complex, leading to some suggesting substitution of the variable with a history of cognitive impairment [32].

The model by Holt and colleagues was developed around the same time as the NHFS but did not receive the same level of attention, despite similar predictive qualities [10, 12]. This might be due to a few issues [33]. First, the model lacks a proper name. Second, no follow-up studies were undertaken to validate the model. Last, its many subdivisions and intricate scoring system might preclude use in daily clinical practice, where time is often limited.

The HEMA was developed by us more recently and while initial results were promising, no external validation was undertaken until now [13]. In this current study, the HEMA demonstrates poor predictive performance when compared to both other models, which might be attributed to several factors. A model developed in a cohort with relatively few cases is at risk of containing variables with high impact but rare occurrence, leading to less reliable predictions [34, 35]. The HEMA uses multiple uncommon variables such as in-hospital fracture and pneumonia at admission. Additionally, several well established risk factors used by other prediction models, such as gender and pre-fracture residency, are markedly absent from the HEMA model. In this study, the lesser performance of the HEMA is reflected in inferior discrimination and a lack of fit for both short- and long-term mortality predictions, as well as in its limited ability of identifying low-risk patients.

Recent years have seen an abundance of publications on risk prediction models for mortality in hip fracture patients [28, 32, 36–41]. Although new models are proposed every year, their added benefit to patient care remains unclear, since these models have not yet proven their worth in daily clinical practice [42]. A prediction model is only of value when it can assist patients and caregivers in clinical decision making. To accomplish this, adequate identification of high- and low-risk patients is essential.

Identification of hip fracture patients at high risk of early mortality remains challenging. In our study, the majority of patients in high-risk groups were alive after 1 year. If risk models are to be used for guidance in end-of-life discussions with patients and their families about treatment options and the possibility of palliative care, accurate identification of patients at very high risk of early mortality is crucial. It seems that as of now, however, no risk prediction model is able to identify this small subgroup of patients.

Identifying low-risk patients has been more promising. In our study, the low-risk subgroups of the NHFS and model by Holt et al. consisted of about a third of all patients. After 8 years, their chance of survival was more than two times higher than average. The difference in mortality between risk groups was most prominent in the NHFS, where the 8-year survival rate of low-risk patients was around 50 times higher than that of high-risk patients (72.4% versus 1.4%). Validated long-term predictions for low-risk patients might make models suitable for aiding in clinical decision making, e.g. when considering hemiarthroplasty versus total hip arthroplasty in the treatment of displaced intracapsular hip fractures [43].

There are several limitations to this study. Due to its retrospective nature, the AMTS was not available for our population. The variable was replaced by cognitive impairment, an independent risk factor for mortality in hip fracture patients and well documented in our medical records [32, 44]. Additionally, sample size was limited due to an 18-month period. Small sample size would be particularly detrimental for development of a risk model, however, less so for validation purposes [45]. Strengths of this study include its comprehensive assessment of risk models and their predictive performance. The evaluation of clinical usefulness measures using risk groups provides useful additional information for clinical decision making. Lastly, completeness of long-term survival data allowed for accurate analysis and visualisation using Kaplan–Meier curves.

## Conclusion

To our knowledge, this is the first study examining mortality predictions in hip fracture patients over such an extended period of time. The HEMA demonstrated inferior predictive performance. The NHFS and the model by Holt et al. were reliable prediction models for short- and long-term mortality following a fracture of the hip. Both models allowed for adequate identification of low- and high-risk patients. The NHFS combines accurate prediction with ease of use and has been validated in multiple external validation studies. Further research should focus on the implementation of the NFHS in daily practice and study its ability to improve on clinical decision making.
